# Nutrient Displacement Associated with Replacing Intake of Usual Snacks with Pecans: An Exploratory Analysis of a Randomized Controlled Trial

**DOI:** 10.1016/j.cdnut.2025.107438

**Published:** 2025-04-09

**Authors:** Tricia L Hart, Penny M Kris-Etherton, Kristina S Petersen

**Affiliations:** Department of Nutritional Sciences, Pennsylvania State University, University Park, PA, United States

**Keywords:** nutrient displacement, pecans, snack replacement, ASA24, adults

## Abstract

**Background:**

The effect of instructions to replace usual snacks with nuts on nutrient displacement is unknown.

**Objectives:**

This analysis aimed to investigate nutrient displacement and changes in food pattern component intake that occur with instructions to consume 57 g/d of pecans in place of usual snacks in adults at increased risk of cardiometabolic diseases after 6 wk and 12 wk.

**Methods:**

Data from a 12-wk randomized controlled trial were used for this exploratory analysis. Adults at risk of cardiometabolic diseases were provided with 57 g/d of pecans and instructed to replace habitually consumed snacks with the provided pecans. The control group was instructed to continue consuming their usual diet. Participants completed 3 24-h recalls at baseline, week 6, and week 12 for assessment of nutrient and food pattern component intake. Nutrient displacement was calculated according to a previously described method to estimate the extent to which nutrients provided by pecans displaced nutrients from other foods.

**Results:**

At 12-wk, partial nutrient displacement occurred for energy (84%; 331 ± 47.8 kcal), total fat (55%; 22.6 ± 0.50 g), monounsaturated fat (47%; 10.9 ± 0.23 g), polyunsaturated fat (46%; 5.71 ± 0.07 g), and fiber (60%; 3.29 ± 0.03 g) in the pecan group. Nutrient displacement >100% was observed for carbohydrates (343%; 27.1 ± 0.12 g), and full displacement of saturated fat occurred at week 12 in the pecan group. In the pecan group, intake of nuts and seeds [mean difference 2.3 oz-eq; 95% confidence interval (CI): 1.0, 3.6], oils (19.2 g; 95% CI: 12.6, 25.7), and total protein foods (2.3 oz-eq; 95% CI: 1.0, 3.6) increased. No other differences in food pattern components were observed. Results at week 6 were relatively consistent with those observed at 12-wk.

**Conclusions:**

Instructions to replace usual snacks with pecans resulted in the partial displacement of energy, total fat, unsaturated fats, and fiber. Additionally, pecan consumption increased intakes of food pattern components emphasized in healthy dietary patterns.

This trial is registered at clinicaltrials.gov as NCT05071807.

## Introduction

Nuts are a recommended part of healthy dietary patterns for general health and prevention of many chronic diseases, including cardiovascular disease, which is the leading cause of morbidity and mortality in the United States [1,2]. Additionally, the intake of nuts aligns with the recommendation to replace animal-based protein sources with plant-based sources, and nuts may now carry the recently updated “healthy” nutrient content claim [3,4]. However, nuts are an energy-dense food, and concerns about weight gain with regular intake exist [5]. Evidence from meta-analyses of randomized controlled trials (RCTs) and prospective cohort studies shows that nut intake is not associated with weight gain [5,6]. This could be attributable to the satiety-promoting components in nuts, including protein and fiber [5]. Nuts also require substantial mastication that slows intake and promotes satiation to reduce intake at an eating occasion [7]. Thus, nut intake may reduce food intake from other sources because of reductions in hunger and the desire to eat [8]. Because nuts are a healthy, nutrient-dense food that aligns with dietary recommendations, nuts may be an alternative to commonly consumed nutrient-poor snack foods. In the United States, snacking comprises ∼370 kcal/d [9]. Of concern, an estimated 34% of total added sugars and 20% of SFA are consumed during snacking [11]. Given the satiety-promoting components in nuts, along with recommendations to replace nutrient-poor foods with nutrient-dense alternatives [1], replacing typical snacks with nuts may result in favorable nutrient displacement. The effect of instructions to replace usual snacks with nuts on nutrient displacement is unknown.

Nutrient displacement is defined as the degree to which a supplemented food changes the nutrient composition of a supplemented diet relative to the nutrient composition of the supplemental food [12]. Thus, nutrient displacement examines the extent to which nutrients provided by the supplemental food displace nutrients from nonsupplemental foods included in the diet and offers insight into how individuals implement a food-based intervention. Five previous RCTs have examined the effect of adding nuts to the diet with (k = 1) and without (k = 4) substitution instructions [[12], [13], [14], [15], [16]]. These studies show provision of nuts results in partial energy displacement (19–68%) with a net energy increase (103–222 kcal/d) [[12], [13], [14], [15], [16]]; however, none of these trials showed significant body weight increases with nut intake [[12], [13], [14], [15], [16]]. Prior evidence also demonstrates that nut intake results in partial displacement of total fat [[13], [14], [15], [16]], MUFA [[12], [13], [14],^16^], PUFA, and protein [[12], [13], [14], [15], [16]]. More explicit instructions aligning with guidelines to replace nutrient-poor foods with nutrient-dense nuts would be expected to cause further nutrient displacement because of the removal of foods high in added sugars and saturated fat.

The effect of instructions to substitute usual snacks with pecans on nutrient displacement is unknown. Therefore, the aim of this analysis of data from a previously conducted RCT is to investigate the nutrient displacement that occurs with instructions to consume 57 g/d of pecans in place of usual snacks in adults at increased risk of cardiometabolic diseases after 6 wk and 12 wk. In addition, changes in food pattern component intake were examined to identify shifts in food intake that may explain the observed nutrient displacement. This is an important research inquiry because although nut consumption is recommended, many consumers may require guidance on how to incorporate nuts into a healthy dietary pattern. It was hypothesized that the pecan group would exhibit full energy displacement because of the substitution instructions provided. It was expected that the pecan group would have a higher intake of nuts, seeds, and oils along with a lower intake of added sugars and solid fats, reflecting the replacement of typical snacks with pecans, compared to the usual diet group. This evidence could inform the implementation of dietary recommendations for nut intake and nutrient-dense snacking.

## Methods

### Trial design

This is an exploratory analysis using data from a previously conducted 12-wk, single-blinded, 2-arm parallel-design RCT. The trial methodology and prespecified primary and secondary outcomes have been reported previously [17]. Briefly, 138 adults at increased risk of cardiometabolic diseases were randomly assigned in a 1:1 ratio to the pecan group or the usual diet group. The pecan group was provided with 57 g/d of pecans and instructed to eat these in place of the usual snacks consumed. The control group was instructed to continue consuming their usual diet and provided with grocery store gift cards ($30/mo), equal to the value of the provided pecans, to purchase their typical snacks. Participants provided informed consent, and all procedures were approved by The Institutional Review Board at the Pennsylvania State University. The trial is registered at clinicaltrials.gov (NCT05071807).

### Participants

Participant recruitment and eligibility criteria have been described previously [17]. Briefly, adults between 25–70 y with overweight or obesity [BMI (in kg/m^2^) 25–40] and ≥1 metabolic syndrome feature (i.e., waist circumference ≥94 cm males or ≥80 cm females; triglycerides ≥1.7 mmol/L; high-density lipoprotein cholesterol ≤1 mmol/L males or ≤1.3 mmol/L females; systolic blood pressure ≥130 mmHg or diastolic blood pressure ≥85 mmHg; fasting plasma glucose ≥5.6 mmol/L) [18] were included. Individuals with cardiovascular, liver, kidney, or autoimmune diseases, inflammatory conditions, diabetes, following restrictive diets, or taking medication that may impact lipids/lipoproteins, glycemic control, or blood pressure were not eligible.

### Dietary data

Dietary data were collected using the automated self-administered 24-h dietary assessment tool (ASA24)-2020 and -2022. Participants were asked to complete 9 24-h recalls during the trial (3 at baseline, 3 at week 6, and week 3 at endpoint). Recalls that had implausible reported energy intake (i.e., <600 kcal or >4400 kcal for females and <650 kcal or >5700 kcal for males) were reviewed and removed from analyses based on the NIH recommendations [19], which resulted in the exclusion of 1% of recalls. ASA24-2020 and -2022 use the USDA Food and Nutrient Database for Dietary Studies 2015–2016 and 2017–2018, respectively, for nutrient analysis [20]. ASA24-2020 and -2022 use the USDA Food Pattern Equivalents Database 2015–2016 and 2017–2018, respectively, to assess the intake of food pattern components [20,21].

### Estimation of nutrient displacement

**FIGURE 1:**
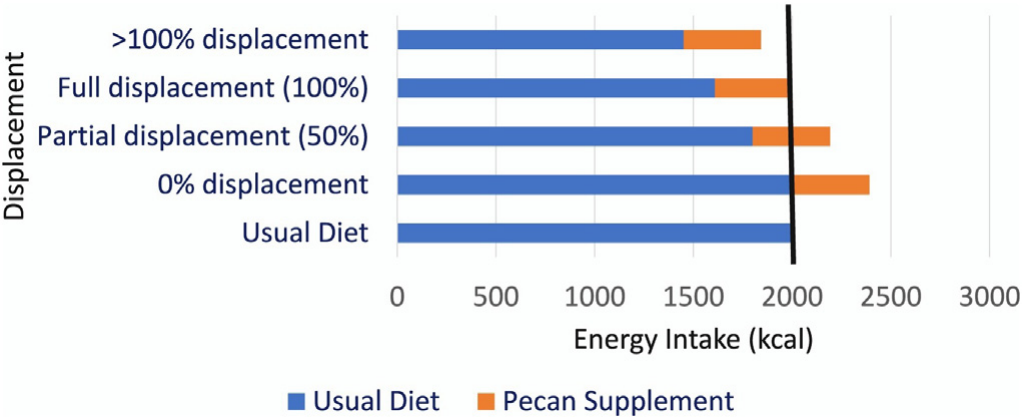
The Concept of Nutrient Displacement. This nutrient displacement example focuses on energy but applies to all nutrients. The pecan supplement provides 394 kcal and is denoted in orange. The base diet is denoted in blue. Greater than 100% displacement results in a net reduction in energy intake. Full displacement occurs when 394 kcal is removed from the base diet and replaced with 394 kcal from pecans. Partial displacement indicates that some non-pecan foods were removed (<394 kcal), resulting in a net increase in energy. 0% displacement implies that participants did not remove any foods from the diet and consumed pecans in addition to their usual diet, leading to a greater net increase than partial displacement. The usual diet serves as a reference, representing a diet devoid of nuts.

Energy and nutrient displacement were calculated according to a previously described method [[12], [13], [14], [15]]. For each participant, the mean of all reliable recalls at week 6 and week 12 was used for analysis. For the nutrient displacement calculation, the intake of the pecan group represents the intake of pecans as part of the usual diet. Intake of the usual diet group represents dietary intake without pecans. The expected intake of the pecan group was calculated by summing the nutrient intake of the usual diet group and the nutrient composition of 57 g of pecans based on the Food and Nutrient Database for Dietary Studies 2019–2020 [22]. Therefore, the expected intake of the pecan group reflects pecans being added to the diet without the removal or displacement of other foods. Nutrient displacement was calculated by subtracting the actual intake of the pecan group from the expected intake of the pecan group. The nutrient displacement percentage was calculated by dividing displacement (expected pecan group - actual pecan group) by the nutrient composition of the provided pecans and multiplying the result by 100. The nutrient displacement percentage was used to estimate the extent to which nutrients provided by pecans displaced nutrients from non-pecan foods. The concept of nutrient displacement is further detailed in Figure 1.

### Statistical analysis

Statistical analyses were performed in SAS (version 9.4; SAS Institute Inc). Nutrient and food pattern component data from reliable dietary recalls were averaged per person per timepoint. Descriptive data used to assess nutrient displacement were evaluated for skewness via Q-Q plots and histograms. Data were approximately normally distributed and are presented as mean ± SE.

Linear mixed models were used to evaluate between-group food pattern component intake changes. Timepoint (baseline, week 6, and week 12) was included as a repeated effect, and group (pecan or usual diet) was included as a fixed effect. The models were adjusted for baseline value, age (years), sex (male or female), and BMI. If a significant group by timepoint interaction was present, post hoc testing was conducted, and the Tukey-Kramer method was used to adjust for multiple comparisons. Group-by-sex interactions were evaluated to assess sex differences; no sex differences were observed, so this interaction term was removed from the final models. The normality of the residuals was evaluated using Q-Q plots, and all data were approximately normally distributed. Compound symmetry was used as the covariance structure for all models based on model fit assessed by the lowest Bayesian information criteria. Significance was set at *P* < 0.05.

## Results

### Participants

Participant characteristics are described in detail elsewhere [17]. Briefly, 138 participants were randomly assigned, with 69 in the pecan group (mean ± SD; 46 ± 12 y; BMI 29.7 ± 3. 7 kg/m^2^; 65% female) and 69 in the usual diet group (46 ± 14 y; BMI 29.8 ± 3.9 kg/m^2^; 52% female). The groups had similar baseline characteristics and self-reported adherence to the intervention/control instructions (instructions were followed on >90% of study days) [17]. The proportion of total daily solid fat and added sugar intake consumed during snacking occasions was similar between the groups at baseline (Supplemental Table 1). The analytical sample at week 6 included 65 participants in the pecan group and 68 in the usual diet group. Sixty-three participants from the pecan group and 68 participants from the usual diet group completed the 12-wk protocol. Weight increased at week 12 in the pecan group [mean difference (MD) 0.70 kg; 95% confidence interval (CI) 0.04, 1.36] compared to the usual diet group [17].

### Displacement of energy and nutrients

Partial energy displacement (absolute displacement ± SE: 363 ± 48 kcal) occurred in the pecan group at week 6 (Table 1). Approximately 92% of the energy from pecans (394 kcal) displaced non-pecan energy sources, resulting in a net energy increase. Nutrient displacement calculations showed that pecan intake resulted in partial displacement of total fat (absolute displacement ± SE: 28.7 ± 0.5 g; 70%), MUFA (absolute displacement ± SE: 12.6 ± 0.2 g; 54%), PUFA (absolute displacement ± SE: 7.1 ± 0.1 g; 57%), and fiber (absolute displacement ± SE: 2.5 ± 0.0 g; 46%). In the pecan group, nutrient displacement >100% was observed for carbohydrate (absolute displacement ± SE: 25 ± 0.1 g; 313%), SFA (absolute displacement ± SE: 7.2 ± 0.0 g; 205%), and protein (absolute displacement ± SE: 7.7 ± 0.0 g; 146%).

**TABLE 1:** Displacement of nutrients after 6 wk- and 12 wk of replacing usual snacks with 57 g pecans in adults at risk for cardiometabolic disease^1^.

	Pecan group (*n* = 65)^2^	Usual diet group (*n* = 68)^2^	Pecans as a snack (57 g)^3^	Expected pecan diet	Displacement	SE displacement	Mean percentage difference (%)	SE percentage difference
Calculation				Usual diet + pecan supplement	Expected pecan diet - pecan group		Displacement/pecan supplement∗100	
Week 6^4^								
Energy, kcal	2144	2113	394	2507	363	47.8	92.1	12.1
CHO, g	218.60	235.50	7.92	243.4	24.8	0.12	313	1.46
Protein, g	83.58	86.01	5.23	91.24	7.66	0.04	146	0.72
Total Fat, g	103.90	91.61	41.0	132.6	28.7	0.50	70.0	1.21
MUFA, g	41.37	30.63	23.3	53.93	12.6	0.23	53.9	0.97
PUFA, g	25.85	20.60	12.3	32.90	7.05	0.07	57.3	0.61
SFA, g	28.43	32.13	3.52	35.65	7.22	0.03	205	0.85
Fiber, g	21.05	18.07	5.47	23.54	2.49	0.03	45.5	0.49
Week 12^5^								
Energy, kcal	2158	2095	394	2489	331	47.8	84.0	12.1
CHO, g	216.7	235.9	7.92	243.8	27.1	0.12	343	1.46
Protein, g	82.22	88.45	5.23	93.65	11.4	0.04	220	0.73
Total Fat, g	107.0	88.58	41.0	129.6	22.6	0.50	55.1	1.21
MUFA, g	42.10	29.70	23.3	53.00	10.9	0.23	46.8	0.97
PUFA, g	26.40	19.81	12.3	32.11	5.71	0.07	46.4	0.61
SFA, g	31.08	31.07	3.52	34.57	3.49	0.03	99.7	0.85
Fiber, g	20.31	18.17	5.47	23.60	3.29	0.03	59.8	0.49

Abbreviations: CHO, carbohydrate; MUFA, monounsaturated fatty acid; PUFA, polyunsaturated fatty acid; SE, standard error; SFA, saturated fatty acid; USDA, United States Department of Agriculture.

1 Statistical analyses were performed with SAS version 9.4 (SAS Institute).

2 Mean of reliable 24-h recalls at week 6 and week 12.

3 Data for pecan nutrients (57 g) was calculated using data from FoodData Central, SR legacy database, 2019, USDA Agricultural Research Service.

4 Data derived from 65 participants in the pecan group and 68 in the usual diet group.

5 Data derived from 63 participants in the pecan group and 67 in the usual diet group.

Results were similar at 12 wk (Table 1). Partial nutrient displacement occurred for energy (absolute displacement ± SE: 331 ± 47.8 kcal; 84%), total fat (absolute displacement ± SE: 22.6 ± 0.5 g; 55%), MUFA (absolute displacement ± SE: 10.9 ± 0.2 g; 47%), PUFA (absolute displacement ± SE: 5.7 ± 0.1 g; 46%), and fiber (absolute displacement ± SE: 3.3 ± 0.0 g; 60%). At week 12, nutrient displacement >100% occurred for carbohydrates (absolute displacement ± SE: 27.1 ± 0.1 g; 343%) and protein (absolute displacement ± SE: 11.4 ± 0.0 g; 220%). At week 12, complete displacement (∼100%) of SFA occurred in the pecan group (i.e., all SFA that was removed from non-pecan sources was replaced by the SFA in pecan), meaning SFA intake was equal to that of the usual intake group.

### Food pattern component changes

At 6 wk, the pecan group had a higher intake of total protein foods (MD: 2.7 oz-eq; 95% CI: 1.4, 3.2), nuts and seeds (MD: 2.6 oz-eq; 95% CI: 2.1, 3.1) and oils (MD: 17.2 g; 95% CI: 10.7, 23.4) than the usual diet group (Table 2). Results were consistent between week 6 and week 12. At week 12, the pecan group continued to consume more total protein foods (MD: 2.3 oz-eq; 95% CI: 1.0, 3.6), nuts and seeds (MD: 2.8 oz-eq; 95% CI: 2.3, 3.3), and oils (MD: 19.2 g; 95% CI: 12.6, 25.7) compared to the usual diet group. No other food pattern component differences were observed between the groups.

**TABLE 2:** Model-based estimates for between-group mean differences in intake of food pattern components in adults at increased risk for cardiometabolic diseases^1^.

Food group	Pecan			Usual diet			Effect^2^
	Baseline *n* = 69	Week 6 *n* = 65	Endpoint *n* = 63	Baseline *n* = 69	Week 6 *n* = 68	Endpoint *n* = 67	
**Total fruit, cup-eq**	1.0 ± 0.1	0.9 ± 0.1	0.8 ± 0.1	0.9 ± 0.1	0.9 ± 0.1	0.8 ± 0.1	0.0 (–0.3, 0.3)
Fruit juice	0.2 ± 0.0	0.1 ± 0.0	0.1 ± 0.0	0.1 ± 0.0	0.2 ± 0.0	0.1 ± 0.0	–0.1 (–0.2, 0.1)
Whole fruit	0.8 ± 0.1	0.8 ± 0.1	0.7 ± 0.1	0.8 ± 0.1	0.7 ± 0.1	0.6 ± 0.1	0.1 (–0.2, 0.4)
**Total vegetables, cup-eq**	2.0 ± 0.1	1.9 ± 0.1	1.9 ± 0.1	2.1 ± 0.1	1.8 ± 0.1	1.8 ± 0.1	0.1 (–0.4, 0.5)
**Total grains, oz-eq**	6.4 ± 0.2	6.1 ± 0.2	6.2 ± 0.3	6.6 ± 0.2	7.0 ± 0.2	7.0 ± 0.2	–0.8 (–1.8, –0.2)
Whole grains	1.0 ± 0.1	0.8 ± 0.1	0.7 ± 0.1	1.0 ± 0.1	1.0 ± 0.1	1.0 ± 0.1	–0.3 (–0.7, 0.2)
Refined grains	5.4 ± 0.2	5.4 ± 0.3	5.4 ± 0.3	5.6 ± 0.2	6.0 ± 0.2	6.0 ± 0.2	–0.6 (–1.6, 0.5)
**Total protein foods, oz-eq**	6.4 ± 0.3^a^	8.4 ± 0.3^b^	8.2 ± 0.3^b^	6.5 ± 0.3^a^	5.7 ± 0.3^a^	6.0 ± 0.3^a^	2.3 (1.0, 3.6)
Meat, poultry, seafood	4.8 ± 0.3	4.9 ± 0.3	4.4 ± 0.3	5.0 ± 0.3	4.5 ± 0.3	5.0 ± 0.3	–0.6 (–1.8, 0.6)
Eggs	0.7 ± 0.1	0.6 ± 0.1	0.7 ± 0.1	0.7 ± 0.1	0.8 ± 0.1	0.6 ± 0.1	0.1 (–0.2, 0.4)
Nuts and seeds	0.8 ± 0.1^a^	2.9 ± 0.1^b^	3.0 ± 0.1^b^	0.7 ± 0.1^a^	0.3 ± 0.1^c^	0.2 ± 0.1^c^	2.8 (2.3, 3.3)
**Total dairy, cup-eq**	1.7 ± 0.1	1.5 ± 0.1	1.6 ± 0.1	1.6 ± 0.1	1.9 ± 0.1	1.6 ± 0.1	0.0 (–0.4, 0.4)
Milk	0.5 ± 0.1	0.4 ± 0.1	0.5 ± 0.1	0.5 ± 0.1	0.5 ± 0.1	0.6 ± 0.1	–0.1 (–0.3, 0.1)
Yogurt	0.1 ± 0.0	0.1 ± 0.0	0.1 ± 0.0	0.1 ± 0.0	0.1 ± 0.0	0.1 ± 0.0	0.0 (–0.1, 0.1)
Cheese	1.0 ± 0.1	0.9 ± 0.1	1.0 ± 0.1	1.0 ± 0.1	1.2 ± 0.1	0.9 ± 0.1	0.1 (–0.2, 0.4)
**Oils, g**	31.2 ± 1.6^a^	45.4 ± 1.6^b^	46.8 ± 1.6^b^	31.3 ± 1.6^a^	28.3 ± 1.6^a^	27.6 ± 1.6^a^	19.2 (12.6, 25.7)
**Solid fats, g**	38.9 ± 1.8	35.2 ± 1.8	37.4 ± 1.8	39.5 ± 1.8	42.9 ± 1.8	40.3 ± 1.8	–2.9 (–10.3, 4.4)
**Added sugars, tsp**	12.5 ± 0.7	11.4 ± 0.7	11.3 ± 0.7	12.3 ± 0.7	13.0 ± 0.7	13.0 ± 0.7	–1.7 (–4.7, 1.3)

Abbreviations: CI, confidence interval; SE, standard error.

1 Statistical analyses were performed with SAS version 9.4 (SAS Institute). Linear mixed models were used to determine the within-and between-group mean difference. When a significant main effect of the group-by-visit was detected, post hoc testing was conducted. Results of post hoc testing are denoted with superscript letters. Values within a row without a common letter differ; Tukey adjusted *P* < 0.05. Data are presented as least-squares means ± SE.

2 Between-group mean difference at endpoint (Tukey adjusted 95% CI).

## Discussion

Instructions to replace usual snacks with pecans resulted in partial displacement of energy, total fat, PUFA, and MUFA, resulting in net increases in intake at 12 wk. The estimated net energy increase (63 kcal) observed at 12 wk approximately aligns with the weight gain observed in the pecan group after 12 wk. In week 6, the displacement of carbohydrates, protein, and SFA was >100%, meaning the pecan group reduced their intake of carbohydrates, protein, and SFA from non-pecan food sources. In week 12, only carbohydrates and protein exhibited displacement of >100%. The pecan group increased the intake of total protein foods, nuts, seeds, and oils at week 6 and week 12 compared to the usual diet group, which reflects pecan intake. However, no between-group differences in added sugars and solid fats were observed. Reductions in added sugars and solid fats were expected because of typical snacking patterns in United States adults [23]. Taken together, instructions to substitute usual snacks with pecans did not result in complete energy displacement as intended.

Findings of this exploratory analysis suggest that at both 6-wk and 12-wk, energy from non-pecan foods was reduced to compensate for dietary inclusion of pecans. This replacement was not isocaloric, which aligns with results from previous RCTs examining energy displacement in response to supplemental nut intake [[12], [13], [14], [15], [16]]. However, previous trials showed less energy displacement (19–68%) than we observed (92%) [[12], [13], [14], [15], [16]]. This is likely related to the substitution instructions provided in our trial; substitution instructions were not provided in 4 out 5 of the other trials [[12], [13], [14],^16^]. Four of the 5 studies had nonsignificant weight gain (0.4–0.9 kg) trends over a 2–6-mo period, which is similar to the magnitude of weight gain observed in our trial [12,[14], [15], [16]]. The fifth trial was conducted in older adults over 2 y, and nonsignificant weight loss (–0.4 kg) was observed, which the authors attributed to reduced intake with age [13]. The partial energy displacement observed in our study would be expected to result in weight gain.

Our estimates suggest that supplemental pecans resulted in a net increase in energy intake of ∼30 kcal/d at week 6 and ∼60 kcal/d at week 12. These energy estimates approximately align with the weight increase (∼0.7 kg) observed in the pecan group compared with the usual diet group over the 12-wk trial. At baseline, our participants consumed ∼80 kcal less (286 kcal) from snacks compared to estimates of United States adults (370 kcal) based on nationally representative data [9]. Thus, incorporating ∼390 kcal/d of pecans as a snack(s) increased energy intake and led to a positive energy balance. Although it is well established that self-reported energy intake is prone to measurement error that varies by population, the energy estimates we obtained in this study were relatively consistent with the observed weight changes [24]. A validation trial of the USDA automated multiple-pass method in adults with overweight/obesity showed 5–10 dietary recalls were needed to assess within-subject changes and 12–15 recalls were needed to assess between-subject variance in energy and macronutrients over a 6-mo period [25]. In our trial, 3 recalls were administered at each time point; therefore, it is likely that we obtained more accurate energy estimates than previous trials. Thus, despite the substitution instructions that were intended to prevent a net increase in energy intake, increased energy intake and modest weight gain were observed in our trial.

Partial nutrient displacement of total fat (55%), MUFA (47%), PUFA (46%), and fiber (60%) occurred in the pecan group, which resulted in a net increase in intake of these nutrients. Similarly, incorporating supplemental walnuts, hazelnuts, and almonds into diets caused partial displacement of total fat (6–58%) [[13], [14], [15], [16]], MUFA (16–77%) [[12], [13], [14],^16^], and PUFA (4–57%) [[12], [13], [14], [15], [16]]. The variation in results is likely related to the differing fatty acid profiles of the nuts studied and the quantity of nuts provided in each trial (28–75 g) [[12], [13], [14], [15], [16]]. However, the partial displacement of PUFA and MUFA when incorporating nuts into the diet is relatively consistent across studies [[12], [13], [14], [15], [16]].

Nutrient displacement calculations showed that participants reduced carbohydrates, protein, and SFA from non-pecan foods after 6 wk of incorporating pecans into the diet; at 12 wk, the intake of carbohydrates and protein was reduced. Results were similar at week 12, but notably, participants had higher SFA consumption at week 12 compared to week 6. The change in SFA displacement suggests that dietary incorporation of pecans may have varied over the course of the trial. Participants may have incorporated the pecans in different ways (e.g., added to meals) as the study progressed, possibly related to fatigue of the instruction to eat only pecans as a snack. This may have resulted in fewer snack replacements or participants choosing different foods as the trial progressed.

We expected a reduction in intake of added sugars and solid fat with the substitution instructions provided based on data about the nutrient composition of snacks consumed by United States adults [10,11]. At baseline, nutrient analysis of snacks showed that participants in the usual diet group consumed ∼17% of total daily solid fat and added sugar from snacks. The pecan group reported consuming a median of 14.9% of total solid fat and 21.7% of total added sugar from snacks. These data suggest that our participants consumed less solid fat and added sugars from snacks than mean estimates for United States adults (∼20% SFA and ∼34% added sugar from snacks), which may explain why we did not observe reductions in added sugars and solids fats with this intervention [11]. Further, dietary changes and education are likely needed to reduce added sugar and solid fat intake. Despite the stability of added sugar and solid fat intake, improvement in fatty acid consumption resulted in improved adherence to the 2020–2025 Dietary Guidelines for Americans [17].

A strength of this exploratory analysis is that dietary data were captured at 2 post-randomization time points to identify changes that occurred over the 3-mo period. Three 24-h recalls were completed at each timepoint to account for day-to-day variability; however, random error is still likely present and impacted the statistical power. The primary limitation of this exploratory analysis is that dietary intake was captured by self-reported 24-h recalls. The National Cancer Institute reported that 24-h recall estimates of energy intake in Western populations typically underestimate energy intake by 3–34% based on comparative studies, reviews and trials utilizing intake biomarkers [[26], [27], [28], [29], [30]]. In our study, reported energy at the group level aligned with observed weight changes, suggesting limited bias.

## Conclusion

Providing 57 g/d of pecans with instructions to consume them in place of usual snacks for 12 wk resulted in a net reduction in intake of carbohydrates and protein and a net increase in intake of energy, total fat, PUFA, and MUFA. Food pattern component analysis showed that pecan intake increased intake of nuts and seeds, total protein foods, and oils compared to continuing usual intake in adults at increased risk for cardiometabolic diseases. Providing instructions to replace usual snacks with pecans caused the partial displacement of energy and unsaturated fats, leading to an improved fatty acid profile and increased intake of food pattern components that are aligned with a healthy dietary pattern compared to continuing usual intake.

## Author contributions

The authors’ responsibilities were as follows– KSP, PMK-E: designed research and critically reviewed the manuscript; TLH: conducted research and drafted the manuscript; TLH, KSP: analyzed and interpreted the data; KSP: had primary responsibility for final content; and all authors: read and approved the final manuscript.

## Data availability

Data described in the manuscript and analytic code will be made available upon request.

## Funding

This study was funded by the American Pecan Council. The American Pecan Council had no role in the study design, data collection, analysis, or interpretation of the data. The project described was supported by the National Center for Advancing Translational Sciences, NIH, through grant UL1 TR002014. The content is solely the responsibility of the authors and does not necessarily represent the official views of the NIH.

## Conflict of interest

K.S.P and P.M.K-E received a grant from the American Pecan Council to conduct this research. T.L.H reports no conflicts of interest.
