# Supplementary material for: Nutrient Displacement Associated with Replacing Intake of Usual Snacks with Pecans: An Exploratory Analysis of a Randomized Controlled Trial
Source: Curr Dev Nutr. 2025 Apr 9;9(5):107438. doi: 10.1016/j.cdnut.2025.107438 (PMC12083908; doi:10.1016/j.cdnut.2025.107438)
Supplement: Multimedia component 1 [file mmc1.docx]

**Supplementary Material**

**Supplementary Table 1. Proportion of total daily solid fat and added sugar intake consumed during snacking occasions by timepoint and group^1^**

| **Group** | **Timepoint** | **Nutrient** | **n^2^** | **Percent of Total^3^**  **Median (IQR)** |  | **Percent of Total^3^**  **Mean±SD** |
| --- | --- | --- | --- | --- | --- | --- |
| **Usual Diet** | Baseline | Solid Fats | 69 | 17.2 (3.4, 28.5) |  | 21.5±24.8 |
|  |  | Added Sugar | 68 | 17.7 (2.2, 35.2) |  | 24.4±25.0 |
|  | Week 6 | Solid Fats | 68 | 18.0 (5.5, 36.4) |  | 22.4±21.1 |
|  |  | Added Sugar | 67 | 33.8 (10.7, 53.7) |  | 33.7±26.0 |
|  | Week 12 | Solid Fats | 67 | 21.2 (7.6, 21.2) |  | 22.5±17.3 |
|  |  | Added Sugar | 66 | 29.4 (11.6, 46.6) |  | 32.2±25.4 |
| **Pecan** | Baseline | Solid Fats | 68 | 14.9 (7.9, 22.7) |  | 17.0±4.5 |
|  |  | Added Sugar | 69 | 21.7 (8.6, 43.7) |  | 28.7±24.3 |
|  | Week 6 | Solid Fats | 65 | 8.7 (0.0, 8.7) |  | 14.9±15.8 |
|  |  | Added Sugar | 65 | 19.2 (1.1, 39.1) |  | 23.4±22.6 |
|  | Week 12 | Solid Fats | 63 | 11.2 (1.4, 11.2) |  | 14.4±15.0 |
|  |  | Added Sugar | 63 | 29.3 (1.2, 40.0) |  | 26.3±21.4 |

^1^All reliable recalls were averaged per person at each time point and eating occasion (snack or meal). ^2^Number of participants for which a percentage could be calculated. ^3^Percent of total daily solid fat or added sugar intake consumed during snacking occasions
